# Readability of the Written Study Information in Pediatric Research in France

**DOI:** 10.1371/journal.pone.0018484

**Published:** 2011-04-06

**Authors:** Véronique Ménoni, Noël Lucas, Jean-François Leforestier, François Doz, Gilles Chatellier, Evelyne Jacqz-Aigain, Carole Giraud, Jean-Marc Tréluyer, Hélène Chappuy

**Affiliations:** 1 Unité de Recherche Clinique Paris Centre, Hôpital Necker Enfants Malades, Assistance Publique Hôpitaux de Paris, Paris, France; 2 Laboratoire d'Ethique Médicale, Université Paris Descartes, Paris, France; 3 CIC P0901 Mère Enfant, Inserm, Hôpital Necker Enfants Malades, Assistance Publique Hôpitaux de Paris, Université Paris Descartes, Paris, France; 4 CIC E4 Inserm, Hôpital Européen Georges Pompidou, Assistance Publique Hôpitaux de Paris, Université Paris Descartes, Paris, France; 5 Service de pédiatrie, Institut Curie, Université Paris Descartes, Paris, France; 6 Centre d'Investigation Clinique Inserm 9202, Hôpital Robert Debré, Assistance Publique Hôpitaux de Paris, Paris, France; 7 Pharmacologie, Groupe Hospitalier Broca Cochin Hôtel Dieu, Assistance Publique Hôpitaux de Paris, Paris, France; 8 EA3620, Université Paris Descartes, Paris, France; 9 Service d'Urgences Pédiatriques, Hôpital Necker Enfants Malades, Assistance Publique Hôpitaux de Paris, Paris, France; Alberta Research Centre for Health Evidence, University of Alberta, Canada

## Abstract

**Background:**

The aim was to evaluate the readability of research information leaflets (RIL) for minors asked to participate in biomedical research studies and to assess the factors influencing this readability.

**Methods and Findings:**

All the pediatric protocols from three French pediatric clinical research units were included (N = 104). Three criteria were used to evaluate readability: length of the text, Flesch's readability score and presence of illustrations. We compared the readability of RIL to texts specifically written for children (school textbooks, school exams or extracts from literary works). We assessed the effect of protocol characteristics on readability. The RIL had a median length of 608 words [350 words, 25^th^ percentile; 1005 words, 75^th^ percentile], corresponding to two pages. The readability of the RIL, with a median Flesch score of 40 [30; 47], was much poorer than that of pediatric reference texts, with a Flesch score of 67 [60; 73]. A small proportion of RIL (13/91; 14%) were illustrated. The RIL were longer (p<0.001), more readable (p<0.001) and more likely to be illustrated (p<0.009) for industrial than for institutional sponsors.

**Conclusion:**

Researchers should routinely compute the reading ease of study information sheets and make greater efforts to improve the readability of written documents for potential participants.

## Introduction

The participation of minors in clinical research protocols requires authorization from their legal guardians. However, this authorization cannot override the refusal of the child [1], [2]. The investigating pediatrician must therefore seek the child's voluntary cooperation in the research protocol, after providing the child with information appropriate for his or her level of development [3]. European regulations require pediatric patients to be provided with information about studies in which they are asked to participate, including their risks and benefits, in a language that the child is likely to understand [4]. The way in which information is delivered to a child for possible inclusion in a research protocol must be approved by the institutional review board.

Although there is no consensus regarding the use of a separate assent document for research, many institutional review boards require their use when presenting study information for children [5]. Therefore, if required, assent forms should be written and presented in a manner that optimizes understanding. Depending on the age of the child, the information supplied may be provided on an assent form written either exclusively for the child or for both parents and children [5], [6].

The information and consent forms for adults (patients or parents) asked to participate in clinical research studies have been evaluated by several researchers, using readability indices, such as that of Flesch [7]–[18]. Documents for adults are generally long (more than five pages) and of poor readability. Is this also the case for the research information leaflets (RIL) for children? There is no recent publication about the measure of the readability of children's written study information, only for children's health literacy [19]. Most studies in this domain have targeted parents. Only one article to date has reported the readability of written study information for minors [20]. Based on a single RIL, the authors showed that improvements in the readability of this document were accompanied by improvements in both the acceptance of the study and its understanding by children.

The aim of our study was to evaluate the readability of a large sample of pediatric RIL in clinical research and to assess the factors influencing this readability, to determine whether efforts are required to improve the readability of pediatric RIL in clinical research.

## Methods

### Collection of information documents

**Figure 1:**
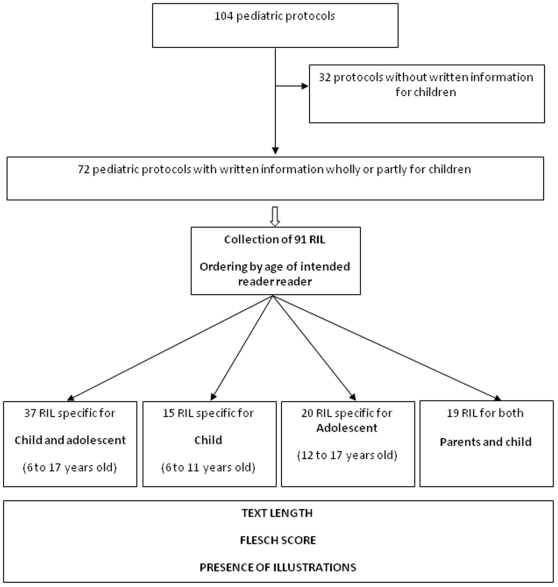
Flow chart of the study. RIL: Research Information Leaflet.

We collected all the pediatric research protocols from three public pediatric clinical research centers in France. All protocols had been authorized by the *Comité de Protection des Personnes* (institutional review board, IRB) between 2002 and 2009. For each protocol, we determined: the goal of the study (therapeutic or not), the type of sponsor (industrial or institutional), the year in which the IRB authorized the study, the field of medical research (oncology or other), whether a randomization procedure was used, whether the disease addressed by the protocol was potentially life-threatening, the phase level of the study (I, II, III or IV) and whether the protocol involved invasive tests (other than taking blood). Pediatric protocols including only children under the age of six years (corresponding to the age at which children learn to read in France) or unconscious children were excluded. RIL for children were collected and classified by age, if the ages of the children to be included were indicated in the inclusion criteria of the protocol or in the document itself. RIL were assigned to four categories on the basis of the age of the intended reader (**figure 1**): child (age between 6 and 11 years), adolescent (age between 12 and 17 years), child and adolescent (age unspecified, 6 to 17 years), and RIL written for parents, to allow them to communicate the necessary information to their children and including a specific space for the child to sign (common parents/child).

### Readability determination

We evaluated readability on the basis of three criteria: length of the text, Flesch readability score and the presence of illustrations [21]. Text length was determined by a word count. Flesch score [22] was calculated as follows: 206.835 - (1.015*sl*) – (0.846*wl*), where *sl* is sentence length (mean number of words per sentence) and *wl* is word length (mean number of syllables per word). The resulting score lies between 0 for texts that are not easily understood, and 100 for readily understandable texts. Scores between 60 and 70 are considered to be the standard reading range for the general population. This score can be calculated with Microsoft Word® software for texts written in English. For texts written in French, we have developed a Flesch score calculator, which is now freely available [21]. The presence of illustrations (pictures, diagrams or tables) was checked manually.

### Comparison texts

Texts appropriate for readers of particular ages were used: extracts from textbooks written for children of 6 to 8 years of age or of 9 to 11 years of age, texts from French national examinations performed in 2000 to 2009 for the *Brevet National des Collèges* targeting 14-year-old adolescents, extracts from children's literature (Harry Potter, Alice in Wonderland, The Little Prince, Pinocchio, Snow White and the Seven Dwarfs and Babar).

RIL for both parents and children were used as comparison texts for RIL written for children, because these texts were mainly targeted at adults.

### Data analysis

The RIL computer files were collected and analyzed with a PERL script that we had previously developed [21]. Statistical analyses were performed with NCSS® software. Continuous variables are presented as medians, with 25^th^ and 75^th^ percentiles. Fisher's exact test was used to compare categorical variables. Nonparametric Mann and Whitney or Kruskal–Wallis tests were used to compare continuous variables, and Tukey-Kramer tests were used for multiple comparisons. A p value <0.05 was considered significant.

## Results

### Protocol population

We included 104 pediatric protocols. Protocols including only patients who could not read, either because they were unconscious (N = 2) or because they were too young (under the age of six years, N = 10) were excluded. We also excluded protocols in which there was no written information for children (N = 20). For the remaining 72 protocols, we collected 91 RIL, which we then classified according to the age of the targeted reader (**figure 1**).

### RIL characteristics

All the data concerning research information leaflet characteristics are included in **table 1**, other than the year in which IRB approval was obtained. Twenty-nine of the RIL (32%) were from oncological studies, whereas the other 62 RIL (68%) encompassed 19 different pediatric specialties: surgical specialties (n = 11, 18%) such as cardiac, orthopedic, ophthalmologic and dental surgery, for example and non surgical specialties (n = 51, 82%), such as infection biology, rheumatology, intensive care, nephrology, diabetology and nutrition for the most part.

**Table 1:** Characteristics of research information leaflets.

	Sponsor		Medical field		Goal		Phase		Randomization		Invasive tests		Vital prognosis	
	Institutional	Industrial	Oncology	Others	Therapeutic	Others	I/II	III/IV	Yes	No	Yes	No	Yes	No
N	75	16	29	62	60	31	26	25	40	51	39	52	48	43
**FLESCH**														
25th Percentile	25	40	23	28	27	26	32	23	28	25	25	28	25	28
Median	**30**	**46**	**34**	**37**	**38**	**30**	**44**	**31**	**38**	**31**	**34**	**38**	**34**	**38**
75th Percentile	42	52	46	46	47	41	52	43	46	44	43	46	45	45
**P value**	0.0005*		0.5832		0.2278		0.012*		0.5277		0.2946		0.2822	
**TEXT LENGTH**														
25th Percentile	355	948	462	320	460	326	487	531	449	358	434	422	447	348
Median	**635**	**1257**	**638**	**841**	**707**	**759**	**754**	**856**	**847**	**635**	**726**	**751**	**624**	**856**
75th Percentile	997	2016	1094	1357	1180	1324	1384	1302	1448	1118	1239	1180	1075	1417
**P value**	<0.0001*		0.7569		0.3633		0.8168		0.0972		0.8580		0.3085	
**ILLUSTRATIONS**														
N	**7**	**6**	**2**	**11**	**13**	**0**	**6**	**2**	**8**	**5**	**7**	**6**	**8**	**5**
%	9	38	7	18	22	0	23	8	20	10	18	12	17	12

### Length of the text

The RIL analyzed comprised a median of 743 words [434; 1211], corresponding to three pages. RIL specifically written for children were significantly (p<0.001) shorter than those written for both parents and children: 608 words [353; 972] versus 1134 [913; 1423] (**table 2**).

**Table 2:** Readability data for 91 research information leaflets used in biomedical research studies, classified by age of the intended reader.

Readability characteristics	Child 6–11 y (group A)	Adolescent 12–17 y (group B)	Child and adolescent 6–17 y (group C)	Common RIL for Parents/Child (group D)
Number of RIL, n (%)	15 (16%)	20 (22%)	37 (41%)	19 (21%)
RIL with illustration, n (%)	7 (47%)	5 (25%)	1(3%)	0
Text length in words, median [25th; 75th percentiles]	607 [464; 869]	1225 [784; 1819]	456 [292; 706]	1134 [836; 1429]
Flesch score, median [25th; 75th percentiles]	46 [38; 52]	40 [30; 47]	37 [27; 43]	25 [22; 28]

Text length and Flesch score also differed significantly between the 4 groups (p<0.001).

An industrial sponsor was the only variable having a significant effect on the length of the RIL (word count). RIL coming from studies having an industrial sponsor were longer (P<0.001) than those from protocols having an institutional sponsor: 1257 [948; 2016] vs. 635 [355; 997]. The other variables studied (year of approval by the IRB, field of medical research, goal of study, phase level, presence of randomization, invasive tests and life-threatening condition) had no effect on RIL length.

### Flesch readability score

**Figure 2:**
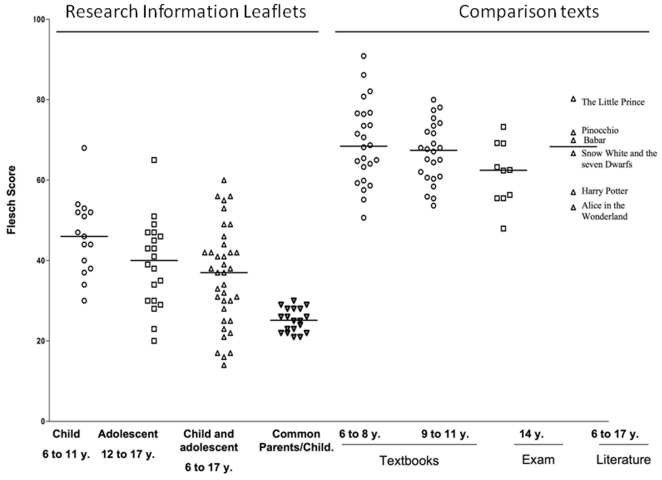
Comparison of the Flesch scores of RIL and comparison texts.

The total Flesch score of the RIL analyzed was 35 [26; 45]. RIL written specifically for children were significantly (p<0.001) more readable than those written for both parents and children: 40 [30; 47] versus 25 [22; 28]. This readability was much lower than that of the texts usually read by children. Textbooks for children aged from six to eight years, books for children aged from 9 to 11 years and extracts from children's literature had Flesch scores of 68 [62; 77], 67 [61; 73] and 68 [55; 76], respectively. French national examination texts used to assess French children's performances in the *Brevet National des Collèges* (a national examination for children aged 14 to 15 years) had a Flesch score of 62 [55; 69]. Pediatric RIL were significantly (p<0.001) less readable than each of these categories of comparison texts (**figure 2**).

Two of the other variables studied were significantly associated with the Flesch score of RIL. RIL from protocols with an industrial sponsor were more readable (p<0.001) than RIL from protocols with an institutional sponsor: 46 [40; 52] vs 30 [25; 42]. RIL from phase I and phase II drug trial protocols were more readable (p<0.001) than RIL from phase III and phase IV drug trial protocols: 44 [32; 52] vs 31 [23; 43].

### Presence of illustrations

Only a small proportion of the RIL analyzed (13/91; 14%) contained an illustration: a drawing to brighten up the document (either linked to the research or purely decorative), a diagram or a table explaining how the study would be carried out, a diagram or photograph of the procedure evaluated in the protocol (medical equipment, surgical procedure). With the exception of the examination papers, all the comparison texts were illustrated (54/64; 84%). RIL from protocols with industrial sponsors were more frequently illustrated than RIL from protocols with institutional sponsors (38% vs 9%; p = 0.009). None of the RIL from non therapeutic protocols was illustrated (p<0.001). Illustrations were present in 23% of the RIL from phase I and phase II trials, versus only 8% of RIL from phase III and IV trials (p = 0.25).

## Discussion

Current regulations [1]–[6] require pediatric patients to be informed, but not necessarily with a written document. However, 78% of our sample of pediatric protocols included the provision of written study information specifically for children. This finding is consistent with those of Whittle *et al.* [6], who interviewed 188 chairpersons of IRBs and reported that 68% of them felt that pediatric information should also be delivered in writing. Similarly, Kimberly [5] showed, by analyzing the decisions of 55 IRBs concerning 69 pediatric protocols, that 83% of the protocols accepted included documentation destined for the child, often divided into two sequential age ranges, each with a different mode of documentation.

Our study is the first to evaluate the readability of RIL from various sponsors as a function of the type of sponsor, aim of the study and risks. The initial sample of 104 protocols was fairly representative of pediatric biomedical research in France. Indeed, on September 27^th^ 2010, a search of the Clinicaltrials.gov site found 270 ongoing interventional pediatric protocols in France. Our study included 40% of these protocols.

Based on our three criteria — Flesch score, length of the text and presence of illustrations — the RIL readability was poorer than that of other French texts destined for children. Several publications have dealt with the readability score of health documents for English, but not with complete pediatric RIL. Most child health information was written at a level above that appropriate for tenth grade [19].

RIL for both parents and children and most of the RIL for children alone contained no illustrations. Only 13 illustrations were identified and they varied considerably in type, as indicated in the results section. We are not aware of any study demonstrating illustrations to be useful in themselves in information documents for pediatric clinical studies, but many texts for children include illustrations. Textbooks and children's literature, which contribute to teaching and education, contained many illustrations, highlighting their importance for a young readership. A good understanding of text often requires is the reader to be able to process elaboratively (i.e., to form vivid mental images of the events of the study). Houts *et al.* [23] showed that the use of pictographs significantly increases the understanding of medical information among patients with low literacy levels.

The widely used Flesch score evaluates the readability of a text as a function of the length of the words and sentences used and has been validated and extensively used for the evaluation of written information readability [8]–[18], [20]–[22]. It therefore facilitates the rapid, objective and quantitative analysis of the complexity of a text. Using it, we found that the texts we selected as comparison texts obtained much higher Flesch scores than RIL, demonstrating the sensitivity of this tool. This score does not reflect the level of patient understanding, because the understanding of any particular individual depends on intrinsic factors (for example, first language, culture, level of education, age etc.). RIL destined for both parents and children, as expected, were less readable than those destined solely for the child. The Flesch index values obtained were very low for information documents destined for both parents and children. The readability of these documents was equivalent to that of documents destined for adults only [21], and was very different from that for texts destined for minors, accounting for the large difference between the values obtained for this type of text and the other categories. There are various issues concerning the ethics of a form destined for both the adult and the child. In addition to the difficulties a child is likely to experience in reading a document written for adults, the information included and the manner of expressing that information should be different in a document intended for children, particularly for the youngest children.

The RIL from phase I and II trials (with an industrial or institutional sponsor) were more readable and more likely to be illustrated than the RIL from phase III and IV protocols. This better readability and presentation may be due to the particular context of phase I and II protocols, in which the evaluation of drug risks is often the main aim of the study and which often include children who have experienced treatment failure. A similar observation was recently made by Cheung *et al.* [24], during an analysis of the readability of informed consent forms for adults.

It is somewhat impractical in the clinical setting to provide multiple assent forms written to satisfy all ages and/or reading abilities. Tait *et al.* [20] demonstrated that a single modified assent form appeared to close the gap in understanding between the younger and older children. This suggests that use of a modified format will be important in providing younger children with developmentally appropriate information that can enhance their decision-making abilities.

The readability of written study information intended for children asked to participate in clinical research was uniformly poor, and much worse than that of the texts usually read by children. Study information is written and presented with little consideration for the literacy, cognitive abilities and preferences of children, and this may be an ethical issue. Researchers should routinely compute the reading ease of study information sheets and make greater efforts to improve the readability of written documents for potential participants. Both the investigators and the IRBs should check these documents to ensure that their readability is appropriate for the age of the children targeted. Flesch scores are thus a potentially useful tool for improving the readability of information documents. Additional work should focus on how best to present information to children so that they are able to choose how much information they require and investigators can learn how best to educate their young potential research subjects.
